# ATG5 overexpression is neuroprotective and attenuates cytoskeletal and vesicle-trafficking alterations in axotomized motoneurons

**DOI:** 10.1038/s41419-018-0682-y

**Published:** 2018-05-24

**Authors:** Tatiana Leiva-Rodríguez, David Romeo-Guitart, Sara Marmolejo-Martínez-Artesero, Mireia Herrando-Grabulosa, Assumpció Bosch, Joaquim Forés, Caty Casas

**Affiliations:** 1 Institut de Neurociències and Department of Cell Biology, Physiology and Immunology, Universitat Autònoma de Barcelona (UAB), Centro de Investigación Biomédica en Red sobre Enfermedades Neurodegenerativas (CIBERNED), 08193 Bellaterra, Barcelona, Spain; 2 Department of Biochemistry and Molecular Biology, Center of Animal Biotechnology and Gene Therapy (CBATEG), UAB, CIBERNED, Barcelona, Spain; 3 Hand and Peripheral Nerve Unit, Hospital Clínic i Provincial, Universitat de Barcelona, Barcelona, Spain

## Abstract

Injured neurons should engage endogenous mechanisms of self-protection to limit neurodegeneration. Enhancing efficacy of these mechanisms or correcting dysfunctional pathways may be a successful strategy for inducing neuroprotection. Spinal motoneurons retrogradely degenerate after proximal axotomy due to mechanical detachment (avulsion) of the nerve roots, and this limits recovery of nervous system function in patients after this type of trauma. In a previously reported proteomic analysis, we demonstrated that autophagy is a key endogenous mechanism that may allow motoneuron survival and regeneration after distal axotomy and suture of the nerve. Herein, we show that autophagy flux is dysfunctional or blocked in degenerated motoneurons after root avulsion. We also found that there were abnormalities in anterograde/retrograde motor proteins, key secretory pathway factors, and lysosome function. Further, LAMP1 protein was missorted and underglycosylated as well as the proton pump v-ATPase. In vitro modeling revealed how sequential disruptions in these systems likely lead to neurodegeneration. In vivo, we observed that cytoskeletal alterations, induced by a single injection of nocodazole, were sufficient to promote neurodegeneration of avulsed motoneurons. Besides, only pre-treatment with rapamycin, but not post-treatment, neuroprotected after nerve root avulsion. In agreement, overexpressing ATG5 in injured motoneurons led to neuroprotection and attenuation of cytoskeletal and trafficking-related abnormalities. These discoveries serve as proof of concept for autophagy-target therapy to halting the progression of neurodegenerative processes.

## Introduction

Connectivity is needed for neuronal survival. Disruption of synaptic function and axonal connectivity precedes neuronal cell death in most neurodegenerative processes and diseases^1^. Accordingly, proximal axotomy leads to dysfunction, atrophy, and eventually neuronal death. Injured neurons may trigger endogenous mechanisms of neuroprotection that help them to rapidly recover from the insult. Macroautophagy (hereafter referred to as autophagy) and the unfolded protein response activated against the endoplasmic reticulum (ER) stress are examples of these mechanisms. Often neurodegeneration appears concomitantly with anomalies in these corrective mechanisms preventing complete recovery. Correction or potentiation of these endogenous mechanisms of neuroprotection might ensure success of neuroprotective therapy^2^.

Here, we used a non-transgenic model of spinal motor neurodegeneration based on surgical peripheral nerve root avulsion (RA) for axonal connectivity disruption. Spinal motoneurons (MNs) are located throughout the central nervous system (CNS) and send out their axons through peripheral nerves. This positioning facilitates experimental access to soma and axons. The model is commonly used to evaluate neuroprotective strategies for use in treatment of peripheral nerve trauma^2^.

After axotomy, a neuronal retrograde response is initiated. The intensity and time course of this response are influenced by the distance from the axonal injury site, the age, and the animal species, among other factors. In contrast to neonatal MNs^3^, in the adults, axonal transection does not lead to MN death unless the injury is in close proximity to the perikaryon as occurs after root mechanical traction or avulsion (RA)^4–6^. In contrast, after distal axotomy, MNs engage endogenous mechanisms that allow them to recover and even regenerate axons. Thus, we sought to characterize the molecular programs activated after distal axotomy that differ from the neurodegenerative process after RA using unbiased proteomics^7^ with the goal of determining which programs should be activated to ensure neuroprotection. In this study, we demonstrate that selective autophagy^8^ is an important endogenous neuroprotective mechanism engaged after distal axotomy. Since the autophagy process is necessary for MN survival and regeneration, we identified concomitant programs that may contribute to its failure with the goal of establishing a neuroprotective approach for RA.

## Results

### Impaired autophagy flux and aberrant lysosomal protein glycosylation early after RA

**Fig. 1:**
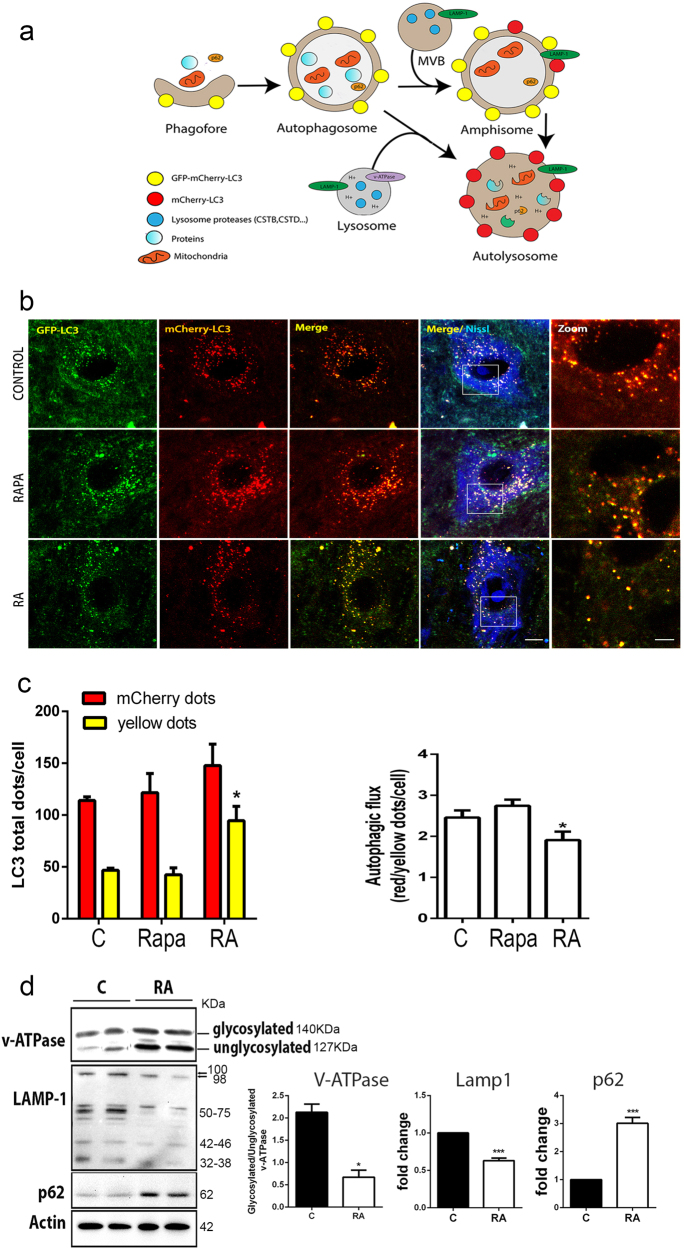
Impaired autophagy flux and aberrant lysosomal protein glycosylation after RA. **a** Schematic representation of the autophagy flux (modified from ref. ^13^). Expression of the mCherry-GFP-LC3 reporter gene will generate yellow puncta upon activation of autophagy at the autophagosome/amphisome and red puncta in the autolysosome if the autophagy flux continues correctly and the lysosomes achieve their usual acidic pH which quenches the green GFP fluorescence. **b** Microphotographs of representative MNs from sham-operated control animals treated with rapamycin (Rapa) for 3 days before sacrifice, from RA injured and control sham rats at 5 dpi; all rats were infected with AAVrh10-mCherry-GFP-LC3. **c** Left, Bar graph of manual quantification of yellow and red puncta within MNs at L4 spinal cord segment in both conditions. Right, Bar graph of autophagic flux measured as the ratio of red to yellow puncta per MN. Non-parametric *t*-test was used to compare the statistical significance between the control and avulsed animal. **d** Left, Immunoblot for glycosylated and unglycosylated v-ATPase, LAMP1, and p62. Note the slight difference in electrophoretic mobility of higher LAMP1 band between control and RA samples and the different general band pattern. Right, Bar graph showing the average fold change protein level ± SEM in control (C) and root avulsed (RA) samples normalized to actin levels (*n* = 4). Scale bar = 10 µm; zoom = 5 µm; *p < 0.05 vs. control, ***p < 0.001 vs. control (Student’s *t*-test)

Autophagy sequesters cytoplasm and superfluous or dysfunctional organelles within an expanding phagophore, leading to the formation of the double-membrane autophagosome (Fig. 1a)^9^. We reported previously that autophagy was induced with the accumulation of lipidated form of microtubule-associated protein 1 light chain 3 (LC3II) at 3–5 days after RA^10^. Increasing levels of LC3II might indicate either enhanced conversion of LC3I to LC3II or impaired degradation through lysosomes. To distinguish between these possibilities, we analyzed autophagic flux using the reporter mCherry-GFP-LC3^11^, which was cloned into the MN-specific adeno-associated viral vector serotype AAVrh10^12^. GFP signal quenches into acidic compartments, such as lysosome, whereas mCherry’s persists (Fig. 1a)^13^.

At 3 weeks after vector injection, the animals were subjected to surgical RA and sacrificed at 5 days post injury (dpi). In order to better detect LC3-associated puncta in sham-operated control animals, the mice were treated orally with rapamycin, an inducer of mTOR-dependent autophagy, for 3 days post operation. By confocal microscopy, we observed abundant LC3-positive autophagosomes and autolysosomes within the MN soma (Fig. 1b). We observed an increase in the number of yellow dots per MN in animals subjected to RA compared to control animals (Fig. 1c). Autophagic flux was significantly diminished to 48% of control levels after RA, suggesting a reduction of autolysosome formation or reduced function.

The existence of autophagy flux impairment after RA led us to focus on late events in autophagy related to fusion and autolysosome formation. We investigated the abundance of three lysosomal proteins critical for its function and fusion with the autophagosome: V-type proton ATPase subunit V0 isoform a1 (v-ATPase), indispensable for lysosome internal acidification; LAMP1, a major integral membrane glycoprotein localized to late endosomes and lysosomes and important for autolysosome fusion events^14^; and the polyubiquitin-binding protein p62/SQSTM1 (p62), an autophagic receptor. The H+ pump activity of v-ATPase depends on its glycosylated state^15^. We observed that the ratio of glycosylated to unglycosylated forms was 4-fold lower after RA than in controls at 7 dpi (Fig. 1d). From this time point, neurodegeneration of avulsed MNs is molecularly initiating as we previously reported^7,10^. LAMP1 was detected as a pattern of bands with different antibodies corresponding to different glycosylated isoforms as previously described^16^ (Supplemental Fig. S1). The overall level of LAMP1 (≈100 kDa) was lower in RA samples than controls, and a particular decrease was observed in faster migrating bands (Fig. 1d). Finally, p62 accumulated in the RA model as expected, given that autophagy flux is impaired (Fig. 1d). These results suggest that RA provokes blockage of the autophagy flux and alterations in the glycosylation of some key intramembrane lysosomal proteins.

### RA causes alterations in microtubule-related proteins and deficient protein sorting

**Fig. 2:**
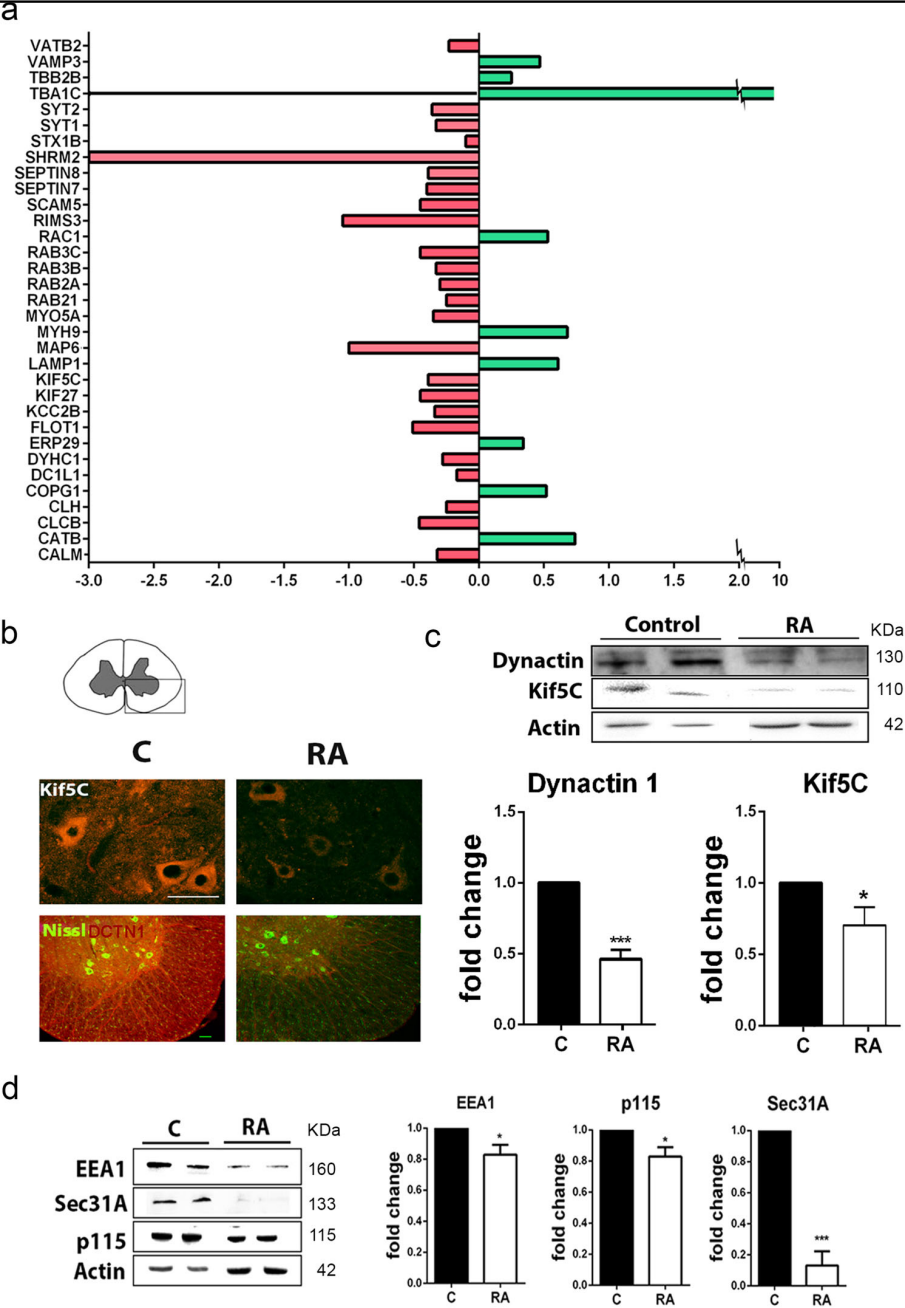
Proteomic data validation. **a** Histogram of microtubule and vesicle-related protein fold changes in RA injured L4–L5 spinal cord segments with respect to sham-operated control obtained by analysis of proteomic data (upregulated in green; downregulated in red) obtained previously^7^. **b** Microphotographs of the ipsilateral spinal cord ventral horn from control (C) and RA-injured (RA) animals, showing immunostaining of KIF5C and DCTN1 with fluorescent Nissl counterstaining to reveal MNs at 7 dpi. Scale bar = 100 µm. **c** Western blot to quantify KIF5C and DCTN1 (*n* = 4; *p < 0.05 vs. control, ***p < 0.001 vs. control Student’s *t*-test). **d** Immunoblot and bar graph showing the analysis of EEA1, Sec31A, and p115 protein levels in L4 spinal cord segments (*n* = 4; *p < 0.05 vs. control, ***p < 0.001 vs. control Student’s *t*-test)

In order to identify the causative events leading to autophagy flux blockage early after RA, we took advantage of our recently performed unbiased quantitative proteomic analysis^7^. From this analysis, we reported a list of protein signatures that determined the neurodegenerative condition. A Gene Ontology (GO) analysis of these data revealed significant enrichment for proteins related to microtubules and vesicle trafficking among those proteins modulated in RA relative to the control (Fig. 2a and Supplemental Table S1). Our proteomic data shed qualitative differences that can be interpreted as quantitative. Qualitative differences depend on both, the easy tripsinization processes for a particular protein and the easy detection of its peptides by the mass spectrometer. Post-translational modifications present on proteins affect easy access for cleavage. Hence, less modified proteins might be easier cut into single peptides and therefore considered as more abundant. This seemed to be the apparent case for LAMP1, which was significantly better detected in RA than in the control group. However, we found less amount of the highly glycosylated 100 kDa band of LAMP1, in agreement with previous reports^10^, and an altered glycosylation pattern in the RA group compared to control. Thus, proteomic data were consistent with an increase in deglycosylated forms.

We also validated other proteins related to either anterograde/retrograde transport, such as the kinesin family protein KIF5C and the dynactin subunit DCTN1, or the secretory pathway. Both KIF5C and DCTN1 diminished significantly after RA compared to control at 7 dpi (Fig. 2b, c). Similarly, there was a reduction in EEA1, at early endosomes, and Sec31A and p115 *cis*-Golgi markers (Fig. 2d)^17^. These data suggested that affectation in microtubule-related transport and vesicle trafficking are early events that occur concomitantly with impaired autophagy flux in this neurodegenerative process.

### In vitro model of MN cell death reproduces transport and glycosylation alterations

An understanding of the relationship and causative sequence between microtubule-related and autophagy impairment after RA will help in the establishment of targets for future neuroprotective therapies. Hence, we aimed to model these features in vitro. We first evaluated the response over time of the MN-like NSC34 cells to treatment with 1 μM rapamycin. We evaluated the expression of Beclin1, involved in the initiation of autophagy, the conversion of LC3I to LC3II, and the level of LAMP1. We observed that the level of these proteins progressively increased from 2 to 4 h after rapamycin treatment (Supplemental Fig. S2).

**Fig. 3:**
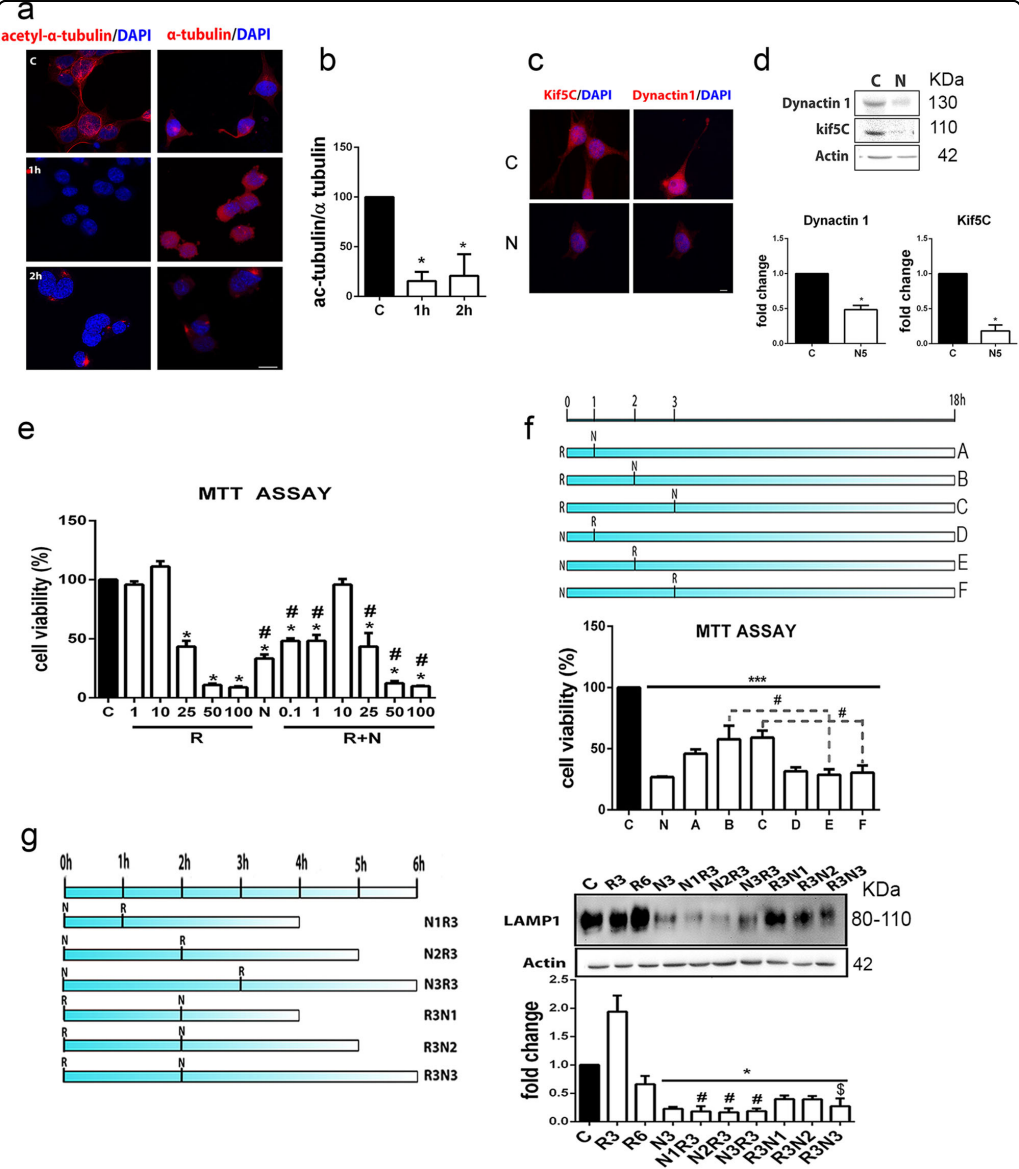
In vitro model characterization. **a** Microphotographs showing merged images for α-acetyl-tubulin and α-tubulin fluorescent immunostaining (red) with DAPI nuclei counterstaining (blue) in control (C) NSC34 cells and cells treated with 10 µM nocodazole (N) for 1 h and 2 h. **b** Bar graph of ratios of mean immunofluorescence intensities (± SEM) of the acetylated vs. non-acetylated forms of α-tubulin (*p < 0.001 vs. control, one-way ANOVA). **c** Representative images of KIF5C and DCTN1 fluorescent immunostaining (red) with DAPI counterstaining (blue) in control cells and cells treated for 5 h with nocodazole. **d** Immunoblots and histograms of the levels of DCTN1 and KIF5C in control and nocodazole-treated cells (*n* = 3; *p < 0.05 vs. control). **e** Bar graph of the mean average percentage of NSC34 cell survival 18 h after vehicle (C), 1 µM rapamycin (R), 10 µM nocodazole (N), and concomitant R+N treatments analyzed by MTT assay (*n* = 4; *p < 0.001 vs. control, one-way ANOVA). **f** Schematic of different culture conditions assayed where either R was added at the beginning of the experiment and N was added after 1, 2, or 3 h (A, B, C conditions) or the reverse (D, E, F conditions). After 18 h of each condition, cell survival was assessed by MTT. **g** Left,  Schematic of culture conditions. N was added at time zero and R was added after 1, 2, or 3 h (N1-N3R3) or the reverse (i.e., R was added at time zero and N was added after 1, 2, or 3 h; R3N1-N3). Cells were harvested at 6 h. Right,  Immunoblot and histogram of LAMP1 protein levels in cells treated as described in **g** (*n* = 3–5; *p < 0.05 vs. control, ^$^*p* < 0.05 vs. R6, ^#^*p* < 0.05 vs. R3, one-way ANOVA)

In order to mimic cytoskeletal alterations, we treated the NSC34 cells with 10 µM nocodazole, which binds to β-III-tubulin and transitorily inhibits microtubule dynamics^18^. Nocodazole treatment caused NSC34 cells to become rounded in shape and drastically reduced the levels of acetylated α-tubulin, KIF5C, and DCTN1 during the first 2 h post treatment compared to control (Fig. 3).

Autophagy induction promoted by rapamycin use did not decrease NSC34 cell survival by MTT assay over 18 h unless used at high concentrations (Fig. 3e). In contrast, despite the transitory effect promoted by nocodazole^7^, it drastically reduced the cell viability (by 41%) (Fig. 3e). To mimic simultaneous induction of autophagy and cytoskeletal alterations as they appear to occur in vivo after RA, the cells were treated with both rapamycin and nocodazole. Co-treatment enhanced cell viability with respect to single treatments at lower concentrations of rapamycin (Fig. 3e). Thus, it is unlikely that microtubule alterations and autophagy occurred at the same time in vivo as we observed MN degeneration.

Since LAMP1 levels are a neurodegenerative signature in our in vivo model, we assayed different sequential treatments with 10 µM nocodazole and 1 µM rapamycin and analyzed the impact on cell survival (Fig. 3f) and on LAMP1 levels (Fig. 3g). We observed that the most drastic effects on cell viability were obtained when nocodazole was added prior to rapamycin, suggesting that microtubule alterations might initiate neurodegeneration after RA. LAMP1 levels and the complexity of the bands observed were dramatically reduced when nocodazole was added prior to rapamycin (Fig. 3g). Considering that the first 2 h of nocodazole treatment should be enough to exert its effect, in subsequent experiments, the cells were first treated with nocodazole; 2 h later rapamycin was added, and analysis was performed 3 h after rapamycin addition (N2R3). Together these results suggest that microtubule alteration precedes autophagy induction after RA to promote neurodegeneration and aberrations in intramembrane lysosomal proteins that compromise lysosomal function.

### Concurrent LAMP1 missorting, lysosomal dysfunction, and blocked autophagy flux in the in vitro model

**Fig. 4:**
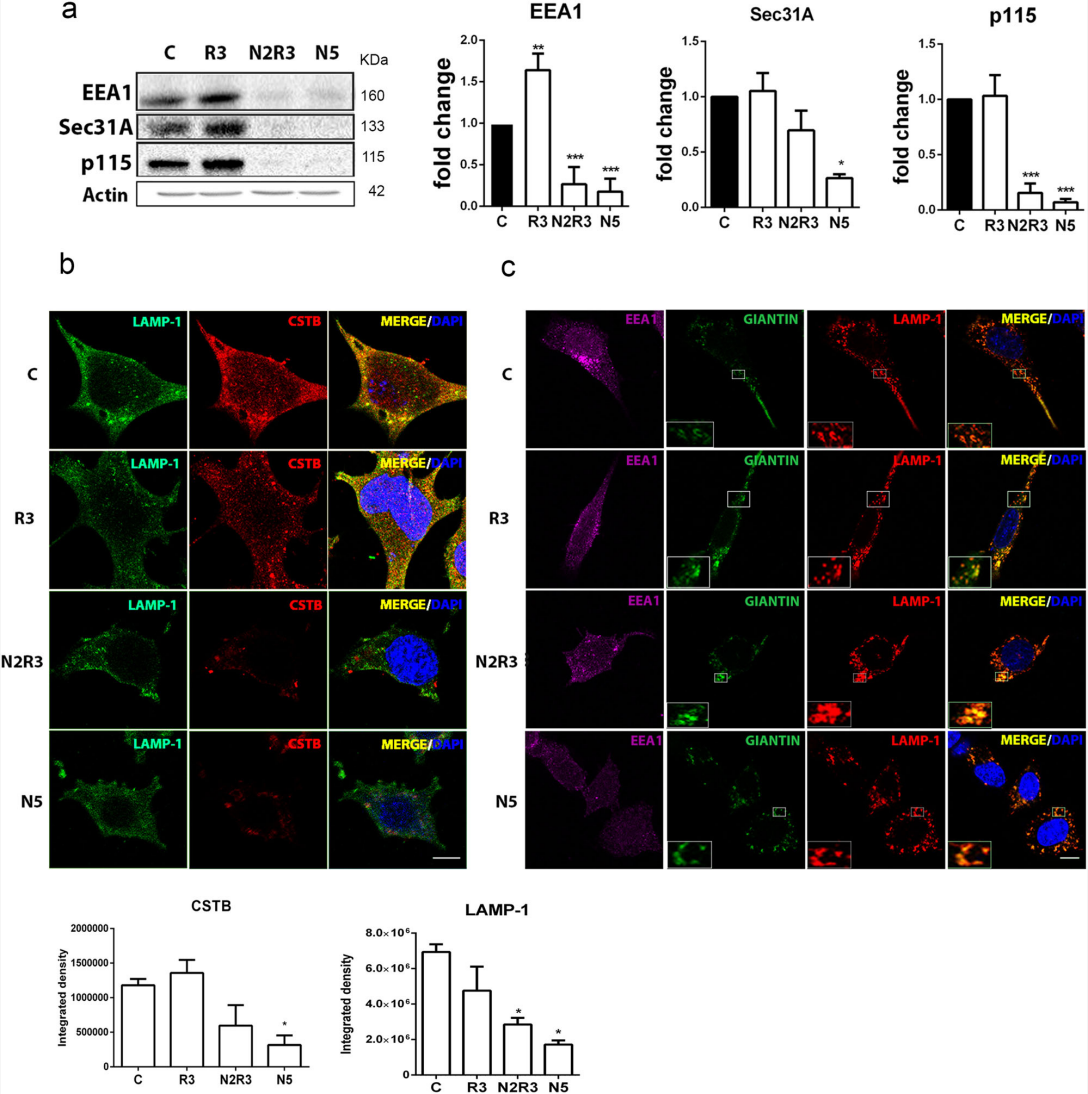
Alterations in protein trafficking in the in vitro model. **a** Immunoblots and histogram showing the levels of EEA1, Sec31A, and p115 in control NSC34 cells and in cells treated with nocodazole (N), rapamycin (R), or the sequential combination (N2R3). **b** Microphotographs showing LAMP1 (green) and CTSB (red) immunostaining and co-localization (merged images) in indicated conditions. Below, graph bars of immunofluorescent quatification for each marker. Scale bar = 15 μm. **c** Representative confocal images of LAMP1 (red) co-immunolocalization (merged) with EEA1 (magenta) and giantin (green). Insets are two-fold magnifications of regions in boxes. Note that LAMP1 and giantin co-localized in cells treated with nocodazole. Scale bar = 30 μm

We suspected that there are alterations in protein trafficking in the secretory pathway specific to the lysosome in the in vitro model and in vivo after RA. By immunoblotting, we observed that the abundances of EEA1, Sec31A, and p115 were drastically reduced 5 h after nocodazole treatment alone or in N2R3 condition (Fig. 4a). Rapamycin alone affected only EEA1 levels. In order to verify that nocodazole treatment altered the lysosomal membrane protein sorting, we analyzed the localization of cathepsin B (CTSB), a lysosome marker, and LAMP1. The proteins did not co-localize in cells treated with nocodazole alone or in combination with rapamycin (N2R3) suggesting impaired trafficking of membrane proteins toward the lysosome (Fig. 4b). In addition, the CTSB-positive lysosomes were located far from the perinuclear position observed in control or rapamycin-treated cells. LAMP1 rather co-localized with giantin (Fig. 4c), a Golgi-marker, distributed into Golgi mini-stacks upon nocodazole treatment^19,20^. This observation verified LAMP1 missorting in the in vitro model.

**Fig. 5:**
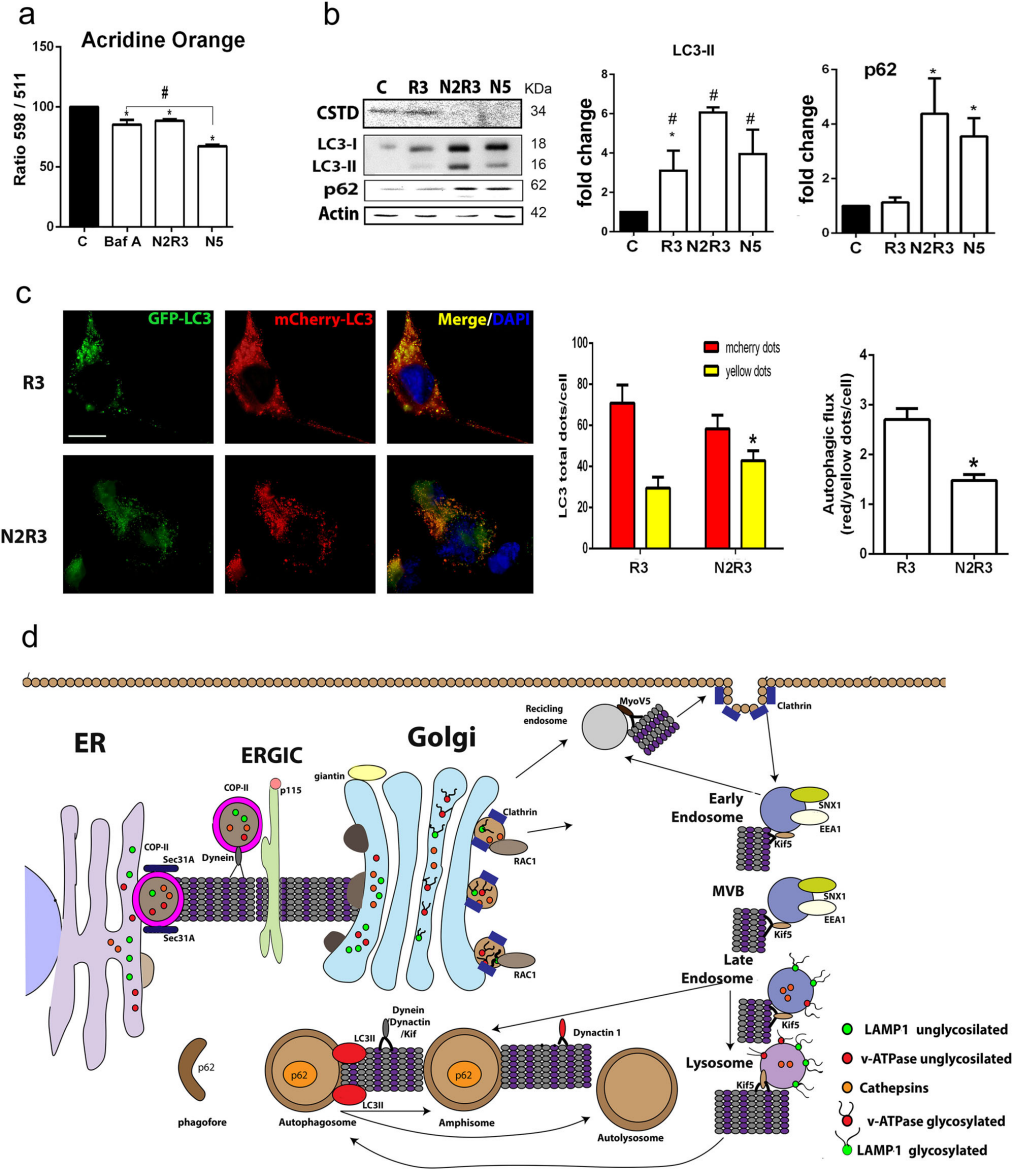
Altered lysosomal function and autophagy flux blockage in the in vitro model. **a** Bar graph of the means (± SEM) of the ratios of the absorbance at 598 (red) to 511 (green) within the cytoplasm of cells stained with acridine orange. Shift toward the green indicates loss of acidity. BafA was used as a positive control (*p < 0.05 vs. untreated control; ^#^*p* < 0.05 vs. nocodazole, one-way ANOVA). **b** Western blot showing the levels of p62, cleaved CTSD, LC3I, and LC3II in control NSC34 cells and cells treated with nocodazole (N) or rapamycin (R) alone or sequentially with nocodazole and rapamycin (N2R3). Bar graphs show the average fold change of these proteins relative to control with normalization to actin. **c** Left, Microphotographs of NSC34 cells transfected with the mCherry-GFP-LC3 plasmid and treated as indicated. Scale bar = 30 µm. Right, Bar graphs with quantification of yellow and red puncta and the ratio (*p < 0.05, non-parametric *t*-test). **d** Schematic of the secretory, endosomal, and autophagy pathways that are affected in MNs early in the neurodegenerative process after RA

We next assessed lysosomal acidification, which is necessary for proper function, using acridine orange (AO) as a sensor that emits red fluorescence in acidic compartments^21,22^. As a control for the evaluation of the ratio of signal at 498 nm (acid) to signal at 511 nm (basic) fluorescence, we treated the cells with 1 μM bafilomycin A1 (BafA1), an inhibitor of v-ATPases^23^, for 3 h. The ratio of acidic to basic signal in BafA1-treated cells was reduced by 15.7±4.2% compared to untreated cells (Fig. 5a). Treatment with nocodazole also decreased the acidity of the cytoplasm (by 11.41% in N2R3 and 32.77% in N5 compared to control cells).

Consequently, we investigated the proteolytic cleavage of cathepsin D (CTSD), which depends on the presence of the acidic pH in the lysosomal compartment. Procathepsin D can escape the transfer to lysosomes and can be secreted into the extracellular space^24^. Hence, in our in vitro system, we could only detect the processed isoform which was drastically reduced after nocodazole, alone or with rapamycin, treatment as expected when acidification of the lysosome is reduced (Fig. 5b). This is in agreement with our previous work reporting altered CTSD processing after RA in vivo^7^.

We next sought to determine whether these alterations were concurrent with autophagy flux impairment in vitro. Western blot analyses revealed that LC3II and p62 accumulated in all conditions that included nocodazole treatment (Fig. 5b). Cells transfected with the reporter LC3-GFP-mCherry had higher numbers of yellow LC3-GFP puncta at the N2R3 condition than when treated only with rapamycin (Fig. 5c). These results suggest that early cytoskeletal alterations severely compromise the function of lysosomes probably due to missorting of important glycoproteins into the lysosomal membrane. This in turn impairs autophagy or other lysosome-related events (Fig. 5d).

**Fig. 6:**
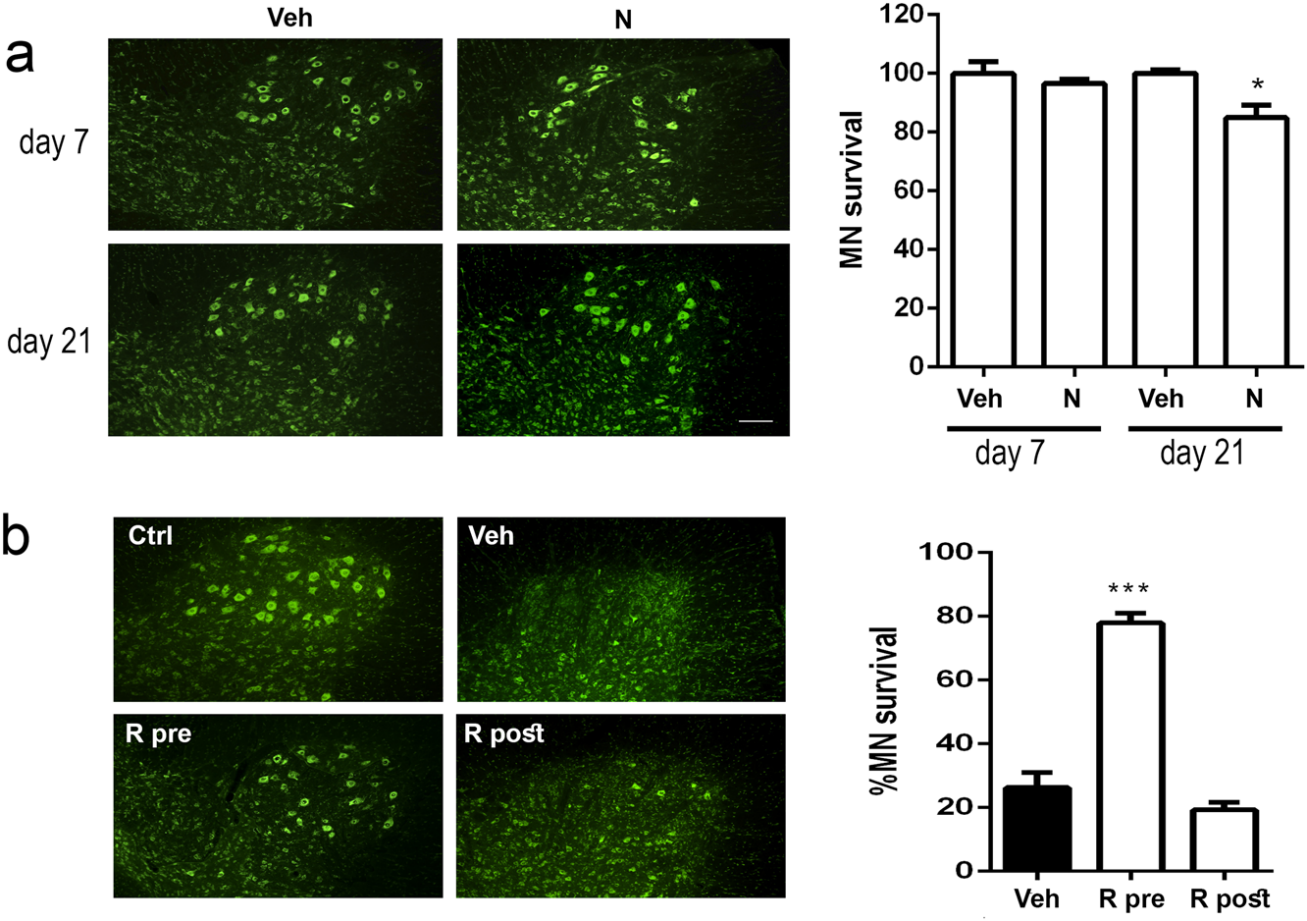
Effects of nocodazole and rapamycin administration in vivo. **a** Left, Microphotographs of fluorescence Nissl stained MNs at the ventral horns of L4–L5 segments of the spinal cord of unlesioned rats treated with a single injection of nocodazole (N) or vehicle (Veh) taken at 7 and 21 days post injection. Right, Bar graphs of the average percentage of MNs at these spinal cord segments compared to control animals at different time-points. **b** Representative images of Nissl stained MNs and associated bar graphs of MN quantification at the ventral horns of L4–L5 segments of spinal cord from animals treated with vehicle (Veh) or rapamycin (R), previously (R pre) or posteriorly (R post) to RA lesion at 21 days post injury (dpi). Scale bar = 50 µm

The early induction of autophagy appears to be necessary for neuroprotection against cytoskeleton-induced alterations, which are also presented after RA. We hypothesized that nocodazole administration would be sufficient to promote neurodegeneration by itself. And also, we wondered if the effect of autophagy induction would yield opposite results depending on pre- or post-treatment to the RA lesion. To explore this, we first intrathecally injected a dose of nocodazole in control rats, without any RA lesion, and let them up to 21 days post injection to analyze MN survival. We observed that nocodazole treatment caused early and transitory reduction of b-tubulin staining of the cytoskeleton as expected (Supplemental Fig. S3a). By the same time, astrogliosis around MNs was significantly increased (Supplemental Fig. S3b). These abnormalities were enough to trigger a slow neurodegenerative process that ends up with a significant decline in the number of MNs by 21 days post injection confirming our initial hypothesis (Fig. 6a). Secondly, we explored whether timing for autophagy induction was crucial for neuroprotection after RA lesions in vivo, as suggested by the in vitro results. On one side, we treated RA-injured animals with rapamycin for 3 days either previously or posteriorly to the lesion. By 21 dpi, similar results were obtained for vehicle administration in pre- and post-treatments. We observed that the number of survived MNs was notably higher in the animals treated with rapamycin previously to the lesion compared to vehicle group. In contrast, no benefit was observed when rapamycin treatment was initiated posterior to the RA lesion (Fig. 6b).

**Fig. 7:**
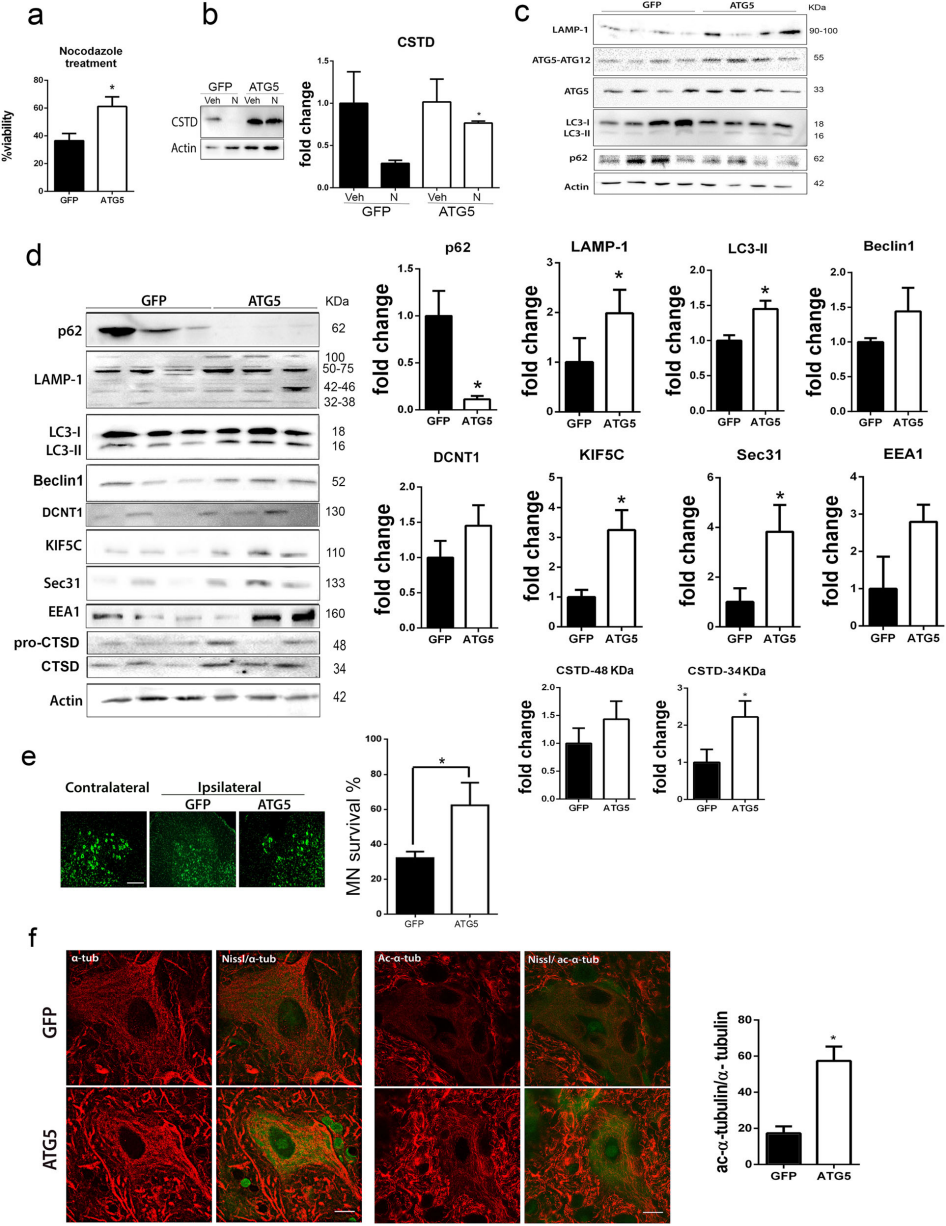
ATG5 overexpression neuroprotects and modulates motor and secretory pathway-related proteins and cytoskeleton. **a** Bar graphs of the mean average percentage of survival of NSC34 cells, transfected with CMV-GFP or CMV-ATG5 plasmids, at 18 h after nocodazole (N) treatment and analyzed by MTT assay (*n* = 4; *p < 0.001 vs. control, one-way ANOVA). **b** Analysis of processed CTSD by western blot in transfected cells treated with vehicle (Veh) or nocodazole. **c** Western blot showing the levels of different autophagy hallmarks at the ipsilateral side of the spinal cord ventral horn from non-injured animals injected with AAVrh10-GFP or AAVrh10-ATG5 at 3 weeks post injection. Note LAMP1, ATG5-ATG12, ATG5, LC3II abundance, and normal p62 levels. **d** Western blot and corresponding bar graphs of the quantification of different proteins related to autophagy (p62, LAMP1, LC3, Beclin1), cytoskeleton (DCTN1, KIF5c), the secretory pathway (Sec31, EEA1), and lysosome function (CTSD) in the spinal cord from RA-injured animals injected with AAVrh10-GFP or AAVrh10-ATG5. **e** Left, Microphotographs showing green fluorescent Nissl stained MNs at the contralateral side and the ipsilateral side of the spinal cord ventral horn from RA-injured animals injected with AAVrh10-GFP or AAVrh10-ATG5. Right, Bar graphs showing the percentage of MN survival at the ipsilateral side with respect to the contralateral side of the same sample. **f** Representative microphotographs of MNs stained with α-tubulin (α-tub) or acetyl-α-tubulin (Ac-α-tub) from the different groups and associated bar graphs of the ratio of the percentage of immunoreactivity for Ac-α-tub with respect to α-tub. Scale bar = 10 µm

Since rapamycin may exert other effects in addition to autophagy induction, we performed a different approach based on autophagy induction by the overexpression of ATG5. In vitro, ATG5 overexpression increased the viability and notably recovered the normal levels of cleaved CTSD forms in nocodazole-treated cells compared to GFP-overexpressing group (Fig. 7a, b). To observe the effects in vivo, we generated the AAVrh10-ATG5 vector and, at 3 weeks after injection, we found (i) a significant overexpression of ATG5 by immunoblotting; (ii) an increased LC3 II isoform, and LAMP1 (≈100 kDa), and (iii) normal p62 levels with respect to animals given the control AAVrh10-GFP vector, as expected for autophagy induction (Fig. 7c). Seven days post RA, ATG5 overexpressing group presented a marked reduction of p62 levels, increase of LAMP1 (≈100 kDa) and LC3-II isoform, and a tendency to rise in Beclin I compared to GFP-overexpressing animals, confirming autophagy induction and proper flux (Fig. 7d). Consistently, we observed increased levels of vesicle trafficking-related proteins such as Sec31 and KIF5C and a tendency to increase in EEA1 and DCTN1 in the AAVrh10-ATG5 compared to the AAVrh10-GFP groups suggesting recovery from a vesicle-trafficking defect after RA (Fig. 7d). By quantifying CTSD forms, we found a significant increase in the cleaved isoform in the ATG5 group suggesting better lysosomal functionality than in the GFP group. In agreement with these results, at 3 weeks post lesion, the number of surviving MNs was higher in the group treated with AAVrh10-ATG5 compared to AAVrh10-GFP-injected animals (Fig. 7e). In addition, the levels of acetyl α-tubulin, a marker of cytoskeletal dynamics^25,26^, were higher in ATG5 with respect to GFP group (Fig. 7f) in agreement with previous results of increased levels in vesicle trafficking-related proteins.

Altogether, these results pointed that early ATG5 overexpression promoted better internal trafficking, autophagy flux, and functionality of the lysosome after RA leading to neuroprotection.

Overall, these results suggested that destabilization of the cytoskeleton is a primary cause sufficient to provoke neurodegeneration of MNs, even if they are still connected, not axotomized, and that primary induction of autophagy, but not late, is a key element for neuroprotection after proximal disconnection by RA.

## Discussion

The nervous system has a remarkable ability for repair under stressful conditions. Under circumstances of damage, intrinsic pro-survival pathways, that are collectively termed endogenous neuroprotective mechanisms, are activated. Here, we addressed the question of why neurodegenerative processes occur even when beneficial mechanisms have been triggered. We confirm that disconnected MNs engage these mechanisms, such as autophagy, but the flux is blocked and anterograde/retrograde transport and secretory and endocytic trafficking are downregulated in vivo due to proximal axotomy. The relevant putative sequences of these events that lead to degeneration are initiated by cytoskeleton-related and vesicle-trafficking abnormalities that cause dysfunction in autophagy flux. We proof that the reverse sequence, i.e., early induction of autophagy, either by ATG5 overexpression or rapamycin pre-treatment, lead to neuroprotection in vitro and in vivo. In particular, precocious autophagy induction by ATG5 overexpression attenuates vesicle trafficking-related abnormalities, normalizes LAMP1 glycosylated pattern, and improves the lysosomal function and microtubule stability. We point to the importance of boosting autophagy at the very early stage in the neurodegenerative process to favor its own proper function and neuroprotection since late induction yields no benefits.

The evidences supporting these conclusions are based on analysis of proteomic data from a non-transgenic model of chronic disconnection by nerve RA (proximal axotomy). This analysis revealed that among the earliest events in the neurodegenerative process were the reduction of cytoskeletal and anterograde/retrograde motor forces (e.g., alterations in tubulins, α-actinin, myosins, kinesins, and dynactin). We previously reported abnormal progressive accumulation of phosphorylated neurofilaments within the soma of avulsed MNs^5^, which is in agreement with defects in microtubules since neurofilaments rely entirely on them for the movement of phosphorylated forms toward the motor axon. KIF5C is a kinesin important for maintaining the MN population in adult mice^27^, implicated in trafficking of mitochondria and vesicles^28^ and in the passage of apical specific cargo molecules, to a post-Golgi endosomal compartment^29,30^. DCTN1 and dynein 1 are part of a multi-protein complex necessary for this trafficking^31^. Proteomic data suggested also a reduction in light and heavy chains of clathrin that may affect clathrin-dependent endocytic machinery or impaired maturation of vesicles emerging from the *trans*-Golgi network. Anterograde/retrograde transport dysfunction due to mutations in kinesins has been reported in patients with Charcot-Marie-tooth type 2A^32^ and in the p150^Glued^ subunit of dynactin in patients with late-onset progressive motor neuron degeneration^33,34^. Transport abnormalities are also observed in the mouse model of the fatal neurodegenerative disease amyotrophic lateral sclerosis (ALS)^1^. Thus, our model of RA reproduces some common key alterations in neurodegenerative conditions affecting MNs.

Correct microtubule dynamics is necessary for protein sorting, and we found alterations in genes encoding proteins involved in the secretory and exocytic pathway (Sec31^35^, sintaxin1, Rac1, and myoVa) as well as the endocytic-lysosomal pathway (clathrin, LAMP1, v-ATPase). The observed reduction of around 90% in the amount of Sec31, which is necessary for the formation of COPII-coated vesicles created at the ER to drive vesicle release toward the *cis-*Golgi^35^, certainly must diminish the transit at the secretory pathway. Mutations in these key components of the secretory pathway (Syntaxin 1b, CALM, CamKII, Rac1, and myoVa) are either lethal or cause severe disease^36–42^.

Membrane proteins from different sources make up the lysosome membrane. Both late-endosomes and lysosomes are highly enriched in distinctive, highly glycosylated and conserved proteins such as LAMP1 that reach their destination directly from the *trans-*Golgi network or from the endocytic pathway. We observed LAMP1 missorting as a consequence of impaired microtubule dynamics in vitro and after RA in vivo^10^. One possible explanation might be saturation of transport at the *trans-*Golgi network since when overexpressed LAMP1 is found at the cell surface^43^. However, we did not observe LAMP1 in the cellular membrane of avulsed MNs in vivo or in nocodazole-treated cells suggesting that saturation is not the cause of missorting. We hypothesize that LAMP1 trafficking is severely reduced at all levels including the step from the ER to the Golgi post RA, resulting in the reduction of the hyperglycosylated forms that we observed both in our in vivo and in vitro models. The underglycosylated form of v-ATPase also accumulated. Absence or reduced function of this pump may prevent lysosomal acidification, explaining the reduction of mature forms of lysosomal enzymes that depend on an acidic environment for maturation, as is the case for CTSD after RA^7^ and in our in vitro model. These observations are in agreement with the findings in other neurodegenerative disease studies: for example, Alzheimer’s disease-linked mutations in presenilins cause reductions in the glycosylation of v-ATPase resulting in defective lysosome-mediated proteolysis during autophagy^44^. Hence, we speculate that this mechanism might be generalized to the neurodegenerative process regardless of specific mutations.

Treatment of cultured MN-like cells with nocodazole reproduced many of the events observed after RA in vivo. Nocodazole and related substances have been used to analyze the role of microtubules in autophagy (reviewed elsewhere^45^). LC3 associates with microtubules, and cells treated with nocodazole or vinblastine, which interfere with microtubule polymerization, are defective in fusion of autophagosomes with lysosomes, but autophagosome biogenesis is not altered in the presence of these drugs^46,47^. It was proposed that nocodazole-induced blockage of autophagic flux is due to the inhibition of autophagosome trafficking toward lysosomes due to impaired microtubule dynamics. This is still a controversial question as in one study microtubule dynamics did not affect the co-localization and fusion of autophagosomes and lysosomes^48^, which has been shown to occur in the absence of microtubules^49^, although others argue that more efficient fusion is enabled by active transport along the microtubules^50^. Two previous studies demonstrated CTSD immaturity due to nocodazole treatment^51,52^. We propose that an altered membrane composition of lysosomes, devoid of proper glycosylated protein forms, in particular LAMP1, due to altered trafficking, contribute to preventing fusion and proper function. Relevant to neurodegeneration from our analysis is that microtubule alterations precede lysosome dysfunction and autophagy flux blockage after RA and hence may prevent induction of neuroprotective programs such as autophagy. Indeed, we provide evidence that a single intrathecal injection of nocodazole in vivo is sufficient to trigger MN degeneration and that autophagy induction before the appearance of any cytoskeletal alteration might prevent MN degeneration. This proof of concept is relevant in the field of neurodegenerative diseases. The targeted neuronal population used to have heterogeneous degrees of pathology, with some of them at late neuropathological stages, while others are still not affected, which gives an opportunity to autophagy induction as a way of halting progression. These results may resolve the questions raised due to controversial studies about the direction in autophagy modulation for therapy, whether being inhibited or activated, regarding some neurodegenerative diseases^53,54^.

Finally, we collect evidence of a possible mechanism by which autophagy induction may prevent the apparition of a neurodegenerative process due to cytoskeletal alterations. By overexpressing ATG5, several issues are correcting: increase in vesicle trafficking-related proteins, improvement of lysosomal function and, importantly, attenuation of microtubule  disorder after the lesion. How might this be possible? One explanation is that the lesion may lead to early aberrant accumulation of cytoskeletal proteins as hyperphosphorylated isoforms^5^. Ready autophagy machinery may help get rid off annoying accumulated material instantaneously before the situation worsens. Therefore, lack of cytoskeletal alterations allows the function of autophagy flux itself as a positive feedback loop.

Thus, enhancing and favoring proper function of the endogenous mechanisms of self-protection, such as autophagy, may be a way to design efficacious neuroprotective strategies to stop progression.

## Materials and methods

### Animal model and drug treatment

Sprague–Dawley female rats aged 12 weeks were kept under standard conditions of light and temperature and fed with food and water ad libitum. We performed surgical procedures under anesthesia with a cocktail of ketamine/xylazine (0.1 ml/100 g weight i.p.) essentially as reported previously^5^. To perform extravertebral nerve RA of the L4–L5 roots, we made a midline skin incision to identify sciatic nerves and applied a moderate traction on selected roots away from the intervertebral foramina, severing the mixed spinal nerves that contained the motor and sensory roots and dorsal root ganglia. The wound was sutured by planes, disinfected with povidone iodine, and the animals were allowed to recover in a warm environment. Sham-operated animals were used as controls. In the experiments for autophagy flux analysis, we administered rapamycin (Sigma-Aldrich) daily into the drinking water at concentrations of 0.2 µM for 3 days, a dose that in control animals favored the visualization of LC3 puncta. For this experiment, treatment of animals started 3 days before the sacrifice. Similarly, to analyze its neuroprotective effect, the animals received rapamycin or 0.1% ethanol as vehicle in the drinking water daily for 3 days either pre- or post-RA surgery. Fifteen microliters containing 50 µM nocodazole (Sigma-Aldrich) or DMSO as a vehicle was injected intrathecally at the dural cistern of L2 the same day of the surgery. All procedures involving animals were carried out in accordance with the guidelines of our institution, and the experimental protocols were approved by the Ethics Committee of our institution, and following the European Community Council Directive 86/609/EEC.

### Autophagy flux analysis

The mCherry-EGFP-LC3B cDNA (kindly provided by Terje Johansen, University of Tromsøl, Oslo, Norway) was cloned into *Nhe*I and *Hind*III sites between the ITR domains of *AAV2* under the regulation of CMV promoter and the woodchuck hepatitis virus responsive element (WPRE)^55^. The AAV2/rh10 vector was generated as previously described^56^ by triple transfection of HEK 293-AAV cells (Stratagene) with branched polyethylenimine (PEI; Sigma-Aldrich) with the plasmid containing the ITRs of AAV2, the AAV helper plasmid containing Rep2 and Cap for rh10 (kindly provided by JM Wilson, University of Pennsylvania, Philadelphia, USA), and the pXX6 plasmid containing helper adenoviral genes^57^. Recombinant vectors were clarified after benzonase treatment (50 U/ml, Novagen) and polyethylene glycol (PEG 8000, Sigma-Aldrich) precipitation. Vectors were purified by iodixanol gradient by the Vector Production Unit at CBATEG (UAB; http://sct.uab.cat/upv) following standard operating procedures. Viral genomes per ml (vg/ml) were quantified using picogreen (Invitrogen)^57^.

Intrathecal administration of viral vector was performed at the lumbar region of isoflurane-anesthetized animals using a 33-gauge needle and a Hamilton syringe. After lateral spine exposure, by paravertebral muscle dissection, 10 µl of viral vectors were slowly injected into the cerebrospinal fluid between vertebrae L3 and L4. Appropriate access to the intrathecal space was confirmed by the animal’s tail movement. The needle was held in place at the injection site for 1 min after which the muscle and skin were sutured. For the analysis, we counterstained sections from rapamycin-treated control and RA-injured untreated rats injected with AAV-mCherry-GFP-LC3 with Fluorescent Nissl Stain (NeuroTrace, Molecular Probes) and analyzed the sections by confocal microscope. Images were acquired with the 63× oil immersion objective using GFP and TXRED filters and merged both channels for co-localization analysis (i.e., red and yellow puncta analysis). Quantitative analyses of LC3B-GFP-mCherry were performed using ImageJ software (National Institutes of Health; available at http://rsb.info.nih.gov/ij/). Image analyses were carried out by selecting the cells in the images and determining the co-localization between GFP and mCherry signals.

### Sample preparation

Rats were deeply anesthetized with dolethal (*n* = 4–5) at 7 days post RA or sham operation to obtain L4–L5 spinal cord segments (5-mm length) for western blot analysis. Samples were snap frozen in liquid nitrogen for storage or were immediately processed by homogenization in lysis buffer (20 mM HEPES, pH 7.2, 250 mM sucrose, 1 mM EDTA, 1 mM EGTA, and a cocktail of protease (Sigma-Aldrich) and phosphatase inhibitors (Roche)) in a Potter homogenizer on ice. After centrifugation of lysates at 800×*g* for 20 min at 4 °C, we collected the supernatant as a cytosolic fraction and quantified the proteins by BCA assay (Pierce Chemical Co.). For western blotting, we loaded 30 μg of cytosolic fractions of L4–L5 segments from each animal onto 12% SDS-polyacrylamide gels to perform electrophoretic separation of the proteins, followed by transference to a PVDF membrane in a BioRad cuvette system in 25 mM Tris, pH 8.4, 192 mM glycine, 20% (v/v) methanol. Membranes were blocked with 5% BSA in phosphate-buffered saline (PBS) plus 0.1% Tween-20 for 1 h at room temperature and then incubated at 4 °C overnight with primary antibody. Antibodies used were the following: anti-β-Actin (A5316; 1:10000; Sigma-Aldrich), anti-ATP6V0A1 (ABIN487206; 1:500; Antibodies Online), anti-DCTN1 (ABIN1683528; 1:500; Antibodies Online), anti-EEA1 (ab50313; 1:1000; Abcam), anti-KIF5C (ab5630; 1:1000; Abcam), anti-LAMP1 (3629; 1:500; Prosci); anti-p62 (610833; 1:100; BD Transduction Laboratories), anti-p115 (612261; 1:1000; BD Transduction Laboratories), and anti-Sec31A (17913; 1:500; Proteintech). After several washes, the membranes were incubated for 2 h with an appropriate secondary antibody conjugated with horseradish peroxidase (1:5000, Vector). The membrane was visualized using a chemiluminescent mixture of one volume 0.5 M luminol, 79.2 mM p-coumaric acid, 1 M Tris-HCl, pH 8.5 and one volume 8.8 M hydrogen peroxide, 1 M Tris-HCl, pH 8.5. Images collected with the Gene Genome apparatus (Syngene) and analyzed with Gene Snap and Gene Tools softwares.

For glycosylation analysis, we used 30 µg protein and submitted to deglycosylation using the Protein Deglycosylation Mix II (New Legends Biolabs) and following the manufacturer’s recommendations before loading the samples in 10% SDS-polyacrylamide.

For immunohistochemistry, at 7 dpi, we transcardially perfused the deeply anesthetized animals with a saline solution containing 10 U/ml heparin followed by 4% paraformaldehyde (PFA) in a 0.1-M phosphate buffer, pH 7.2, for tissue fixation (*n* = 4 at each time post lesion) and removed the L4 and L5 segments (5-mm total length) of the spinal cord, post-fixed in the same fixative for an extra 4 h and cryopreserved in 30% sucrose overnight. We cut the samples into serial transverse sections (20-µm thick) onto gelatinized slides using a cryotome (Leica) and preserved them at −20 °C until used. For immunohistochemistry, we treated the slides with blocking solution in Tris-buffered saline (TBS) with 0.03% Triton-X-100 and 10% bovine serum for 1 h and incubated thereafter with primary antibodies anti-DCTN1 (ABIN1683528; 1:500; Antibodies Online) or anti-KIF5C (ab5630; 1:1000; Abcam). After several washes with TBS, 0.05% Tween-20, the sections were incubated for 2 h with Cy-2- or Cy-3-conjugated donkey anti-rabbit antibodies (Jackson Immunoresearch). We counterstained the sections with DAPI (Sigma-Aldrich), or NeuroTrace Fluorescent Nissl Stain (Molecular Probes) and mounted the slices with Fluoromount-G mounting medium (Southern Biotech) or Mowiol. Sections of injured and control animals were processed in parallel for immunohistochemistry. Images of the ventral horn spinal cord samples were taken under the same exposure times, sensitivities, and resolutions for each marker analyzed with the aid of a digital camera (Olympus DP50) attached to the microscope (Olympus BX51). Confocal microscope examinations were performed with a Zeiss LSM 700 system.

### In vitro model

NSC34 cells were grown in modified Eagle’s medium high-glucose (DMEM) supplemented with 10% fetal bovine serum (Sigma-Aldrich), and 0.5× penicillin/streptomycin solution (Sigma-Aldrich). Cells were kept in a humidified incubator at 37 °C under 5% CO_2_. For the treatments, we coated plastic plates (Thermo) with 10% collagen dissolved in Milli-Q water at 37 °C for 2 h. After removing this solution, we seeded the cells at a density of 2.5×10^5^ per cm^2^. After 4 days of culture without changing the medium, NSC34 cells present with a differentiated-like phenotype characterized by the presence of long neurite extensions. At this time, we added different drugs to the cells. The drugs, prepared at a concentration 10-fold higher than the concentration to be tested, were dissolved in DMEM to the desired concentration and used to replace medium over cells. We used 1 μM rapamycin (Sigma-Aldrich) and 50 μM nocodazole (Sigma-Aldrich) unless otherwise stated. After 18 h, we assessed cell viability by incubating the cells with 0.4 mg/ml of MTT for 3 h. The formed blue formazan crystals were dissolved with DMSO, and the absorbance at 570 nm was measured with a microplate reader (Bio-tek, Elx800) (*n* = 4–5).

We transfected 1×10^5^ cells with 2 µg GFP-LC3-mCherry plasmid (kindly provided by Terje Johansen, University of Tromsøl, Oslo, Norway) using the Amaxa Nucleofector II TM (Lonza) and the Nucleofactor V kit (Lonza) following the manufacturer’s recommendations. LC3 puncta were analyzed as described above.

For western blot, the cells were harvested and homogenized in modified RIPA buffer (50 mM Tris-HCl, pH 7.5, 150 mM NaCl, 1 mM EGTA, 1% NP-40, 0.5% sodium deoxycholate, 0.1% SDS, protease and phosphatase cocktails). For immunocytochemistry, we coated 12-mm glass coverslips with 10% collagen placed in 24-well plates and seeded the cells onto them. After culture, we fixed the cells with 4% PFA, rinsed twice with PBS, and stored at −20 °C or added blocking buffer containing PBS plus 0.3% (v/v) Triton X-100 and 10% fetal bovine serum. We incubated with the following primary antibodies: anti-DCTN1 (ABIN1683528; 1:500; Antibodies Online), anti-giantin (324450; 1:100; Calbiochem), anti-KIF5C (AP52366; 1:100; Acris Antibodies), mouse-anti-α-tubulin (1:500; Sigma Aldrich), mouse-anti acetylated α-tubulin (AA4.3, 1:500; Hybridoma Bank, Iowa City, IA, USA), mouse anti-β-tubulin (1:500, Covance/biolegend, San Diego, CA, USA) or anti-LAMP1; (1D4B, 1:100, Hybridoma Bank) in 0.5× blocking buffer in PBS, at 4 °C overnight. The following day, after several washes with PBS plus 0.05% Tween-20, we incubated the coverslips with Cy3- or Cy2-conjugated secondary antibodies (Jackson Immunoresearch). Coverslips were counterstained with DAPI, and mounted with Mowiol. Images were taken under the same exposure times, sensitivities, and resolutions for each marker analyzed with the aid of a digital camera (Olympus DP50) attached to the microscope (Olympus BX51). Confocal microscope examinations were performed with a Zeiss LSM 700 system. Comparisons for tubulins or GFAP staining were performed taking microphotographs at 20× magnification from at least five spinal cord sections (separated by 200-μm between pairs), and transforming them to gray scale using ImageJ software. Immunoreactivity can be analyzed by measuring the integrated density of a region of interest (ROI) after defining a threshold for background correction 5. The ROIs were selected on the gray matter at the ventral horn and had an area of 0.11 mm^2^. For tubulin immunoreactivity analysis, the ROI enclose the MN soma, determined by Nissl staining. Pictures were obtained for at least 15 MNs extracted from three different sections (separated by 100 μm between each section) per animal for each marker.

### Motor neuron counting

Spinal cord sections were selected with a random start and then sampled systematically (every 12th section) to generate serial subsamples from each lumbar spinal cord of animals at 21 dpi. Eight series of 10 sections (separated by 100 µm) of each processed L4–L5 spinal cord were stained with fluorescent NeuroTrace (Life Technologies, Carlsbad, CA, USA) following the manufacturer’s protocol. Sequential microphotographs were taken covering the lateral ventral horn at 10×. Large MNs were identified by their localization in the lateral ventral horn of lumbar spinal cord sections and only MNs with diameters of 30–70 µm with prominent nucleoli and polygonal shapes located at the layer IX of the ventral horn were counted^2,5,10^. The mean number of MNs per section was calculated. For comparisons, the estimated number of MNs present in the ventral horn of the avulsed side was expressed as a percentage of the contralateral side.

### Analysis of acidic vesicular organelles

To evaluate the acidic vesicular organelles, we incubated cells with AO (Sigma-Aldrich) at a final concentration of 1 μg/ml for 10 min. We pre-treated NSC34 cells with 1 μM of BafA1 (Sigma-Aldrich) to specifically inhibit the vacuolar proton pump v-ATPase mainly at lysosomes. After two washes with pre-warmed PBS with calcium and magnesium, we immediately visualized and analyzed the slices using an Olympus 8160 fluorescence microscope. We collected and recorded the intensity for each excitation wavelength for independent images maintaining the same exposure conditions for all the experiments (*n* = 30 cells/condition). The ratio of 598–511 nm (red vs. green fluorescence) was calculated for each cell and condition.

### Bioinformatics and statistics

We performed GO and pathway analyses of the signatures for degenerative process after RA^7^ with STRING (http://string-db.org/)^58^ and DAVID (http://david.niaid.nih.gov/david/version2/index.htm)^59^ web tools. Data shown are means (±SEM) of a least three independent experiments. We used analyses of variance (ANOVA) to compare the values among different experimental groups for data that met the normality assumption. Difference between groups was analyzed by using a one-way ANOVA, followed by Tukey’s post hoc multiple-range test or Student’s *t*-test. All statistical analyses were done using GraphPad Prism 5 software (*n* = 3–5; *p* < 0.05 for significance).

## Electronic supplementary material

Supplemental figure and table
