# Supplementary material for: ATG5 overexpression is neuroprotective and attenuates cytoskeletal and vesicle-trafficking alterations in axotomized motoneurons
Source: Cell Death Dis. 2018 May 24;9(6):626. doi: 10.1038/s41419-018-0682-y (PMC5967323; doi:10.1038/s41419-018-0682-y)
Supplement: Supplementary file 1 — Supplemental figure and table [file 41419_2018_682_MOESM1_ESM.pdf]

ATG5 Overexpression is Neuroprotective and Attenuates Cytoskeletal and Vesicle-Trafficking Alterations in Axotomized Motoneurons

Tatiana Leiva-Rodríguez[1], David Romeo-Guitart[1], Sara Marmolejo-Martínez-Artesero[1], Mireia Herrando-Grabulosa[1], Assumpció Bosch[2], Joaquim Forés[3], and Caty Casas[1*]

[1] *Institut de Neurociències* and Department of Cell Biology, Physiology and Immunology, *Universitat Autònoma de Barcelona (UAB)*, and Centro de Investigación Biomédica en Red sobre Enfermedades Neurodegenerativas (CIBERNED), 08193 Bellaterra, Barcelona, Spain.

[2] Department of Biochemistry and Molecular Biology, Center of Animal Biotechnology and Gene Therapy (CBATEG), UAB, CIBERNED.

[3] Hand and Peripheral Nerve Unit, Hospital Clínic i Provincial, *Universitat de Barcelona*, Barcelona, Spain.

[*] **Corresponding author:** Caty Casas Louzao, Unitat de Fisiologia Mèdica, Edif. M, Universitat Autònoma de Barcelona, E-08193 Bellaterra, Barcelona, Spain. Tel: +34-935811324, Fax: +34-935812986, E-mail: Caty.Casas@uab.cat

SUPPLEMENTARY INFORMATION

- **Supplementary Tables and Headings**
- **Supplementary Figures and Legends**

SUPPLEMENTARY TABLES AND HEADINGS

Supplementary table S1

Table S1. Fold change of microtubule and vesicle related proteins by proteomics

**GO:0015630 Microtubule cytoskeleton**							
**ID**	**Protein**	**Short Name**	**Fold**	**Std Error**	**t value**	**DF**	**p value**
Q7TP36	Protein Shroom2	SHRM2	-3,04	0,11	-27,97	56	0
P34067	Proteasome subunit beta type-4	PSB4	-1,16	0,14	-8,55	137	4,64E-13
Q63560	Microtubule-associated protein 6	MAP6	-1,00	0,06	-17,85	280	0
P63004	Platelet-activating factor acetylhydrolase IB subunit alpha	LIS1	-0,56	0,07	-8,03	416	2,27E-13
Q9Z1E1	Flotillin-1	FLOT1	-0,51	0,09	-5,49	8	0,00298825
Q7M6Z5	Kinesin-like protein KIF27	KIF27	-0,45	0,10	-4,53	14	0,00252115
P23928	Alpha-crystallin B chain	CRYAB	-0,45	0,11	-3,88	111	0,00108087
B0BNF1	Septin-8	SEPTIN 8	-0,40	0,08	-4,95	37	0,0001273
Q9WVC0	Septin-7	SEPTIN 7	-0,39	0,09	-4,27	164	0,000232
P56536	Kinesin heavy chain isoform 5C	KIF5C	-0,39	0,08	-4,96	15	0,00104572
P62161	Calmodulin OS=Rattus norvegicus	CALM	-0,32	0,09	-3,52	247	0,00263141
P85515	Alpha-centractin	ACTZ	-0,31	0,12	-2,48	203	0,04239646
P38650	Cytoplasmic dynein 1 heavy chain 1	DYHC1	-0,28	0,03	-9,62	2339	0
O55156	CAP-Gly domain-containing linker protein 2	CLIP2	-0,18	0,05	-3,50	132	0,00317932
Q9QXU8	Cytoplasmic dynein 1 light intermediate chain 1	DC1L1	-0,17	0,06	-2,84	175	0,01821581
P63331	Serine/threonine-protein phosphatase 2A catalytic subunit alpha isoform	PP2AA	0,16	0,05	3,28	16	0,01719956
Q3KRE8	Tubulin beta-2B chain	TBB2B	0,25	0,08	3,22	16	0,01915378
Q04631	Protein farnesyltransferase/geranylgeranyltransferase type-1 subunit alpha	FNTA	0,26	0,08	3,39	118	0,00451095
Q02293	Protein farnesyltransferase subunit beta	FNTB	0,34	0,13	2,72	22	0,03921408
Q62812	Myosin-9	MYH9	0,68	0,12	5,87	98	7,03E-07
P31977	Ezrin	EZRI	2,37	0,26	9,25	8	0,0001186
P37397	Calponin-3	CNN3	3,35	0,17	19,63	36	0
Q6AYZ1	Tubulin alpha-1C chain	TBA1C	9,33	0,28	32,95	12	7,87E-12
**GO:0031988 Membrane-bounded vesicle**							
**ID**	**Protein**	**Short Name**	**Fold**	**Std Error**	**t value**	**DF**	**p value**
Q9Z2I6	Synaptic vesicle glycoprotein 2C	SV2C	-0,66	0,25	-2,58	47	0,04016482
P02770	Serum albumin OS=Rattus norvegicus	ALBU	-0,58	0,06	-9,56	548	0
Q63377	Sodium/potassium-transporting ATPase subunit beta-3	AT1B3	-0,57	0,21	-2,73	56	0,02829316
P12785	Fatty acid synthase	FAS	-0,55	0,05	-12,24	1042	0
P06685	Sodium/potassium-transporting ATPase subunit alpha-1	AT1A1	-0,53	0,07	-7,22	312	7,34E-11
Q9Z1E1	Flotillin-1 OS=Rattus norvegicus	FLOT1	-0,51	0,09	-5,49	8	0,00298825
P08082	Clathrin light chain B OS=Rattus norvegicus	CLCB	-0,46	0,06	-7,63	16	9,84E-06
Q9JKE3	Secretory carrier-associated membrane protein 5	SCAM5	-0,45	0,10	-4,77	16	0,00126997
P62824	Ras-related protein Rab-3C	RAB3C	-0,45	0,13	-3,51	53	0,00443394
Q05683	Glutamate decarboxylase 2	DCE2	-0,44	0,18	-2,51	63	0,04413076
P10824	Guanine nucleotide-binding protein G(i) subunit alpha-1	GNAI1	-0,39	0,06	-6,91	38	4,02E-07
P11598	Protein disulfide-isomerase A3	PDIA3	-0,38	0,06	-5,93	378	8,64E-08
P29101	Synaptotagmin-2	SYT2	-0,36	0,10	-3,65	237	0,00179161
Q9QYF3	Unconventional myosin-Va	MYO5A	-0,35	0,07	-5,35	172	2,89E-06
P08413	Calcium/calmodulin-dependent protein kinase type II subunit beta	Camk2B	-0,34	0,10	-3,44	86	0,00436509
Q63941	Ras-related protein Rab-3B	RAB3B	-0,33	0,11	-3,04	31	0,01743794
P21707	Synaptotagmin-1	SYT1	-0,33	0,08	-4,24	120	0,00030561
P04639	Apolipoprotein A-I	APOA1	-0,32	0,09	-3,68	51	0,00286613
Q6P6R2	Dihydrolipoyl dehydrogenase, mitochondrial	DLDH	-0,30	0,07	-4,22	223	0,00025467
P63012	Ras-related protein Rab-3A	RAB3A	-0,29	0,09	-3,05	142	0,01117429
P05712	Ras-related protein Rab-2A	RAB2A	-0,27	0,06	-4,48	190	0,00010494
Q6AXT5	Ras-related protein Rab-21	RAB21	-0,25	0,10	-2,49	51	0,04811081
P13233	2',3'-cyclic-nucleotide 3'-phosphodiesterase	CN37	-0,25	0,06	-3,96	559	0,0005567
P11442	Clathrin heavy chain 1	CLH	-0,25	0,04	-6,46	1787	2,10E-09
P62815	V-type proton ATPase subunit B, brain isoform	VATB2	-0,23	0,07	-3,05	639	0,01005739
Q9QXU9	ProSAAS	PCSK1	-0,20	0,05	-4,12	105	0,0004988
O55156	CAP-Gly domain-containing linker protein 2	CLIP2	-0,18	0,05	-3,50	132	0,00317932
P63039	60 kDa heat shock protein, mitochondrial	CH60	-0,17	0,07	-2,45	645	0,04392197
P47709	Rabphilin-3A	RP3A	-0,16	0,07	-2,49	183	0,04127707
P06761	78 kDa glucose-regulated protein	GRP78	0,15	0,04	3,48	435	0,00280816
Q9QXQ0	Alpha-actinin-4	ACTN4	0,16	0,06	2,94	374	0,01364871
P24368	Peptidyl-prolyl cis-trans isomerase B	PPIB	0,30	0,10	3,08	99	0,01100049
P31044	Phosphatidylethanolamine-binding protein 1	RKIP	0,30	0,11	2,72	228	0,02394697
Q63081	Protein disulfide-isomerase A6	PDIA6	0,31	0,10	3,13	152	0,00878527
Q5XIE0	Acidic leucine-rich nuclear phosphoprotein 32 family member E	AN32E	0,33	0,09	3,73	6	0,03126756
P52555	Endoplasmic reticulum resident protein 29	ERP29	0,34	0,05	6,36	66	2,72E-07
P07153	Dolichyl-diphosphooligosaccharide-protein glycosyltransferase subunit 1	RPN1	0,39	0,07	5,83	88	1,06E-06
P04785	Protein disulfide-isomerase	PDIA1	0,40	0,09	4,39	296	0,00012347
Q8CGU6	Nicastrin	NICA	0,44	0,15	2,96	13	0,03523719
P37361	Metallothionein-3	MT3	0,48	0,16	2,93	20	0,02744153
Q4AEF8	Coatomer subunit gamma-1	COPG1	0,52	0,13	3,86	41	0,00216109
Q6RUV5	Ras-related C3 botulinum toxin substrate 1	RAC1	0,53	0,17	3,22	67	0,00838511
P14562	Lysosome-associated membrane glycoprotein 1	LAMP1	0,61	0,11	5,37	45	2,41E-05
Q91ZN1	Coronin-1A	COR1A	0,63	0,10	6,53	92	4,78E-08
P00787	Cathepsin B	CATB	0,74	0,16	4,60	185	6,78E-05
A8WCF8	Tumor protein p63-regulated gene 1-like protein	TPRGL	0,87	0,30	2,89	37	0,02253646
Q66HD0	Endoplasmin	ENPL	1,20	0,17	7,17	74	6,82E-09
P17475	Alpha-1-antiproteinase	A1AT	1,57	0,07	22,55	109	0
P38659	Protein disulfide-isomerase A4	PDIA4	1,69	0,09	18,91	52	0
Q6AYC4	Macrophage-capping protein	CAPG	1,74	0,10	17,12	101	0
P49134	Integrin beta-1	ITB1	4,55	0,12	37,16	26	0
P06238	Alpha-2-macroglobulin	A2MG	8,08	0,04	188,46	13	0
Q9ESR9	ATP-binding cassette sub-family A member 2	ABCA2	9,76	0,02	406,61	7	0
**GO:0060627 regulation of vesicle-mediated transport**							
**ID**	**Protein**	**Short Name**	**Fold**	**Std Error**	**t value**	**DF**	**p value**
Q9JIR3	Regulating synaptic membrane exocytosis protein 3	RIMS3	-1,046	0,08488	-12,33	5	0,0004234
Q9QWN8	Spectrin beta chain, brain 2	SPTN2	-0,696	0,10844	-6,419	92	7,73E-08
P08082	Clathrin light chain B	CLCB	-0,464	0,06083	-7,628	16	9,84E-06
P08082	Clathrin light chain B	CLCB	-0,464	0,06083	-7,628	16	9,84E-06
Q9JKE3	Secretory carrier-associated membrane protein 5	SCAM5	-0,454	0,09517	-4,767	16	0,00126997
P62824	Ras-related protein Rab-3C	RAB3C	-0,45	0,12808	-3,512	53	0,00443394
P10824	Guanine nucleotide-binding protein G(i) subunit alpha-1	GNAI1	-0,387	0,05606	-6,907	38	4,02E-07
O35964	Endophilin-A2	SH3G1	-0,363	0,09542	-3,804	56	0,00194406
P29101	Synaptotagmin-2	SYT2	-0,363	0,09932	-3,654	237	0,00179161
P61265	Syntaxin-1B	STX1B	-0,354	0,09272	-3,816	336	0,00100266
Q925N3	PEX5-related protein	PEX5R	-0,347	0,10823	-3,203	14	0,0225164
P21707	Synaptotagmin-1	SYT1	-0,33	0,07792	-4,241	120	0,00030561
Q6RJR6	Reticulon-3	RTN3	-0,319	0,05911	-5,398	144	2,88E-06
P63012	Ras-related protein Rab-3A	RAB3A	-0,289	0,0949	-3,049	142	0,01117429
P05712	Ras-related protein Rab-2A	RAB2A	-0,272	0,06084	-4,479	190	0,00010494
P61023	Calcium-binding protein p22	CHP1	-0,263	0,0995	-2,648	45	0,03523719
P11442	Clathrin heavy chain 1	CLH	-0,254	0,03929	-6,464	1787	2,10E-09
Q99N27	Sorting nexin-1	SNX1	0,146	0,05728	2,5422	88	0,03961777
Q62940	E3 ubiquitin-protein ligase NEDD4	NEDD4	0,204	0,0639	3,1972	83	0,00838511
P17077	60S ribosomal protein L9	RL9	0,419	0,13978	2,9957	81	0,01390317
P02650	Apolipoprotein E	APOE	0,438	0,08778	4,9893	183	1,32E-05
P63025	Vesicle-associated membrane protein 3	VAMP3	0,467	0,0646	7,2357	7	0,0010564
Q4AEF8	Coatomer subunit gamma-1	COPG1	0,516	0,1338	3,8565	41	0,00216109
Q6RUV5	Ras-related C3 botulinum toxin substrate 1	RAC1	0,535	0,16598	3,2231	67	0,00838511
P61751	ADP-ribosylation factor 4	ARF4	0,558	0,11304	4,9402	9	0,00397693
Q91ZN1	Coronin-1A	COR1A	0,626	0,09578	6,5329	92	4,78E-08
Q66H80	Coatomer subunit delta	COPD	0,831	0,17141	4,8493	69	6,36E-05

SUPPLEMENTARY FIGURES AND LEGENDS

Supplementary Figure S1

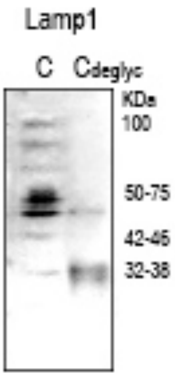

Supplementary Fig. S1. Western blot with anti-Lamp1 using sample of L4-L5 spinal cord extract from a control rat (C) and the same after being deglycosilated for 18h (C_{deglyc}). Note totally shift of band pattern.

Supplementary Figure S2

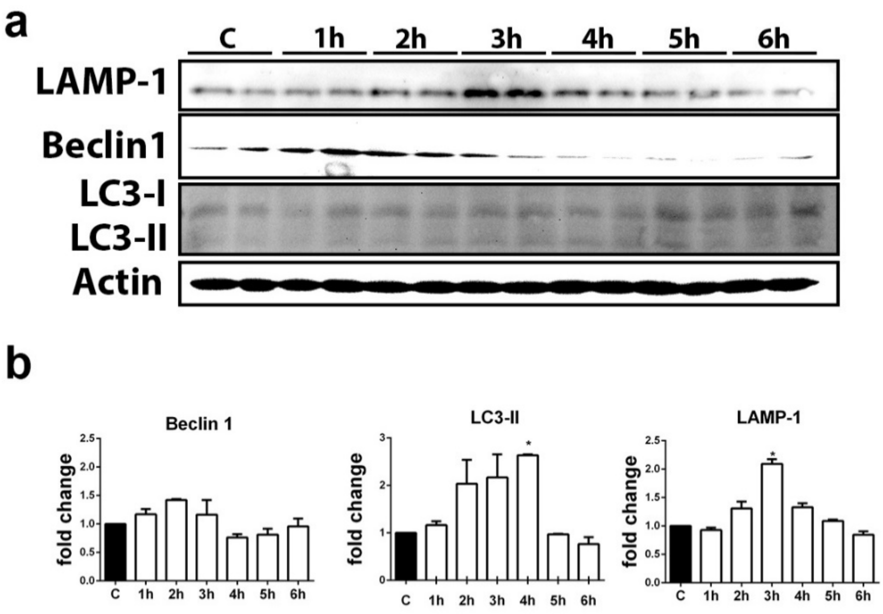

Supplemental Figure S2. Ncs34 cell time-course response to rapamycin treatment. (**a**) Immunoblotting of key autophagy-related molecules after 10 μM rapamycin added to the culture

medium for different time exposition. (**b**) Bar graph of fold change of Beclin, LC3II and LAMP1 at different time-points respect to actin values.

Supplementary Figure S3

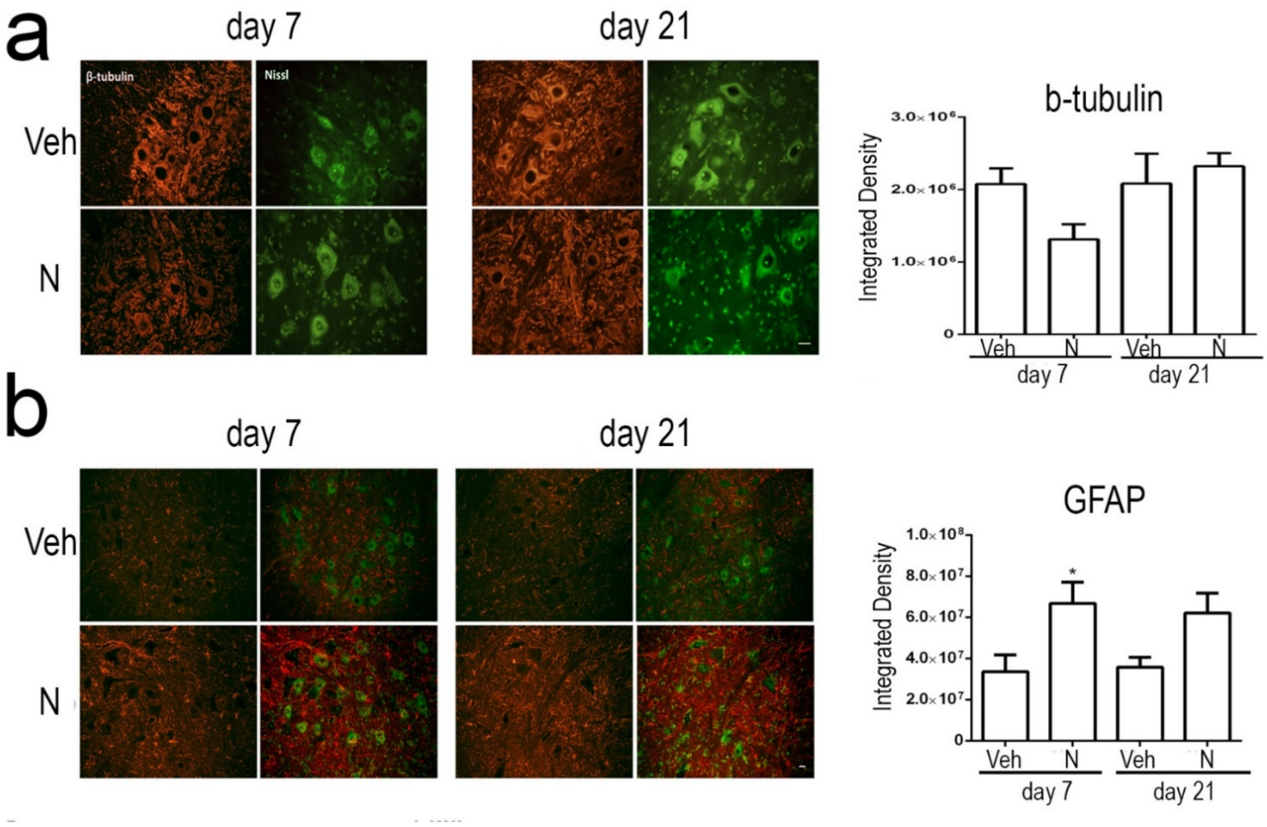

Supplementary Figure S3. (**a**) **Left**, Representative microphotographs of β-tubulin (red) and green fluorescent Nissl of MNs at the ventral horns of the spinal cord from animals receiving either vehicle (Veh) or nocodazole (N) injection at 7 or 21 days post injection. **Right,** Bar graphs of the integrated density for β-tubulin within MNs at the different conditions. (b) Images and bar graphs of immunoreactivity analysis for GFAP staining (red) at the ventral horns with MNs labelled in green (Nissl) at the same conditions described. Scale bar= 25 μm.
